# Whole-Genome Sequence of *Hafnia alvei* HUMV-5920, a Human Isolate

**DOI:** 10.1128/genomeA.00556-16

**Published:** 2016-06-16

**Authors:** María Lázaro-Díez, Santiago Redondo-Salvo, Aroa Arboleya-Agudo, Javier Gonzalo Ocejo-Vinyals, Itziar Chapartegui-González, Alain A. Ocampo-Sosa, Felix Acosta, Luis Martínez-Martínez, José Ramos-Vivas

**Affiliations:** a Servicio de Microbiología, Hospital Universitario Marqués de Valdecilla, Santander, Cantabria, Spain; b Instituto de Investigación Sanitaria Valdecilla (IDIVAL), Santander, Cantabria, Spain; c Red Española de Investigación en Patología Infecciosa (REIPI), Instituto de Salud Carlos III, Madrid, Spain; d Instituto de Investigación en Ingeniería de Aragón (I3A), Universidad de Zaragoza, Zaragoza, Spain; e Servicio de Inmunología, Hospital Universitario Marqués de Valdecilla-IDIVAL. Santander, Cantabria, Spain; f Grupo de Investigación en Acuicultura, Universidad de Las Palmas de Gran Canaria, Arucas, Gran Canaria, Spain; g Departamento de Biología Molecular, Universidad de Cantabria, Santander, Spain

## Abstract

A clinical isolate of *Hafnia alvei* (strain HUMV-5920) was obtained from a urine sample from an adult patient. We report here its complete genome assembly using PacBio single-molecule real-time (SMRT) sequencing, which resulted in a chromosome with 4.5 Mb and a circular contig of 87 kb. About 4,146 protein-coding genes are predicted from this assembly.

## GENOME ANNOUNCEMENT

*Hafnia alvei* is a Gram-negative facultatively anaerobic bacillus that belongs to the family *Enterobacteriaceae*. In humans, it has generally been considered an opportunistic bacterium, causing infections associated with underlying illnesses or predisposing conditions, as in immunocompromised patients (1). Although some virulence traits have been studied in *H. alvei*, little is known about the factors that contribute to their pathogenesis within a host, including adherence, cytotoxicity, biofilm formation, and quorum sensing (2–4). Moreover, recent investigations have focused on associations between the genus *Hafnia* and emerging antimicrobial resistance patterns (5, 6).

The strain used in this study (HUMV-5920) was isolated from the urine sample from a woman at the Hospital Universitario Marqués de Valdecilla in Santander, Spain. The strain was routinely cultured in Luria-Bertani (LB) agar or broth at 37°C and frozen at -80°C with 20% glycerol. This strain produces quorum-sensing signals and forms biofilms. The total genomic sample of *H. alvei* strain HUMV-5920 was extracted and purified using the GeneJET genomic DNA isolation kit (Thermo Scientific). The genomic DNA was submitted to Macrogen (South Korea) for PacBio single-molecule real-time (SMRT) sequencing. A single library was prepared for *H. alvei* HUMV-5920 and run on one SMRT cell. With a genome size of approximately 4.6 Mb, PacBio SMRT sequencing provided approximately 100% coverage of the entire *H. alvei* HUMV-5920 genome. SMRT sequencing initially resulted in 141,257 raw reads, with a mean subread length of 7,457 bp, totaling 1,053,404,563 nucleotides. The generated reads were then introduced into the Hierarchical Genome Assembly Process version 3 (HGAP3), which includes assembly with the Celera Assembler and assembly polishing with Quiver (7). The final complete genome resulted in a circular chromosome of 4,542,863 bp, with a total G+C content of 48.8%, and a circular contig of 87,576 bp, with a total G+C content of 43.6%. A total of 4,146 protein-coding sequences were predicted, of which 22 encode rRNA and 91 encode tRNA. The RAST server (8) predicted coding sequences belonging to 548 subsystems, including 560 involved in carbohydrate catabolism, 280 in protein metabolism, 433 in the synthesis of amino acids and derivatives, 143 in cell wall and capsule synthesis, 214 in RNA metabolism, and 117 in DNA metabolism, including 329 in cofactors, vitamins, prosthetic groups, or pigments, 126 in nucleoside and nucleotide synthesis, 125 in fatty acid and lipid synthesis, 101 involved in virulence, disease, and defense, 179 in membrane transport, 156 in stress response, 60 in phosphorus metabolism, 121 in regulation and cell signaling, 6 in secondary metabolism, 60 phages, and 140 in motility and chemotaxis.

### Nucleotide sequence accession numbers.

The complete genome sequence of *H. alvei* strain HUMV-5920 has been deposited at DDBJ/EMBL/GenBank under the accession numbers CP015379 (chromosome) and CP015380 (plasmid).
